# Spontaneous Abdominal Bleeding from an Infundibulopelvic Ligament Tear in a Patient with Large Ovarian Fibroma

**DOI:** 10.1155/2019/9834915

**Published:** 2019-07-16

**Authors:** Justin To, Cui-lan Li

**Affiliations:** ^1^Division of Minimally Invasive Gynecologic Surgery, Department of Obstetrics and Gynecology, Flushing Hospital Medical Center, 4500 Parsons Blvd, Flushing, NY 11355, USA; ^2^Visiting physician in Department of Obstetrics and Gynecology, Flushing Hospital Medical Center, USA

## Abstract

Ovarian fibromas are rare benign solid tumors of the ovary which are often difficult to differentiate from uterine leiomyomas preoperatively. The symptoms usually include abdominal discomfort and may have ascites and/or an elevation in CA-125 levels. There have been no publications of associated abdominal bleeding to date. The treatment is surgical removal via a laparoscopic or laparotomic approach. We present a case of a 19 cm unilateral ovarian fibroma with abdominal bleeding from a spontaneous right infundibulopelvic ligament (IPL) tear who underwent a laparoscopic and mini-laparotomic right salpingo-oophorectomy. Patients with large ovarian fibromas should be cautioned that abdominal bleeding and/or acute abdominal pain can occur and that a minimally invasive surgical approach is feasible.

## 1. Introduction

Ovarian fibromas are the hardest benign solid tumors that belong to the category of sex-cord stromal cell tumors of the ovary. They account for about 4% of benign ovarian tumors [1]. Ovarian fibromas are usually misdiagnosed preoperatively as uterine fibroids. When ascites is present (Meigs syndrome) with elevated levels of CA-125, they may be misdiagnosed as ovarian malignant tumors or ovarian endometriosis [2]. They generally are diagnosed in the perimenopausal or menopausal period. There are also reports in young patients as well. In these cases, Gorlin syndrome, also known as nevoid basal cell carcinoma syndrome, must be excluded [3].

Symptoms usually include abdominal discomfort or pain. Ascites can be associated, but there have been no reported cases in the literature of associated abdominal bleeding. The treatment for ovarian fibromas is surgical removal via laparoscopy or laparotomy. Son CE et al. [4] reported that the median tumor diameter in their laparoscopic group was 7 cm, whereas the median in the laparotomic group was 8 cm. For giant ovarian fibroma cases, surgeons may be reluctant to use laparoscopic management, as it can be difficult to remove the tumor. We present a case of a 19 cm unilateral ovarian fibroma with abdominal bleeding from a spontaneous infundibulopelvic ligament (IPL) tear who underwent a laparoscopic and mini-laparotomic right salpingo-oophorectomy.

## 2. Case Report

A 39 year-old unmarried gravida 0 para 0 woman presented to the gynecology office complaining of a self-detected right pelvic mass noted 3 years ago which increased significantly in size during the last year. She complained only of mild pressure discomfort. She had no complaints of constitutional symptoms. She had no medical or surgical history. Physical examination showed a non-tender abdomen and a mobile solid hard mass of nearly 20 cm in diameter. Pelvic ultrasonography and MRI reported a 19 x 14 x 12 cm right-sided subserosal leiomyoma of the uterus, and normal bilateral ovaries. There was no sign of ascites, free fluid, or plural effusion and no preoperative tumor markers were obtained. Her pre-operative hemoglobin was 13.2 g/dL and hematocrit was 37.8%. The patient was scheduled for laparoscopic myomectomy and indicated procedures. On the day of surgery, she had no additional complaints.

## 3. Procedure

**Figure 1:**
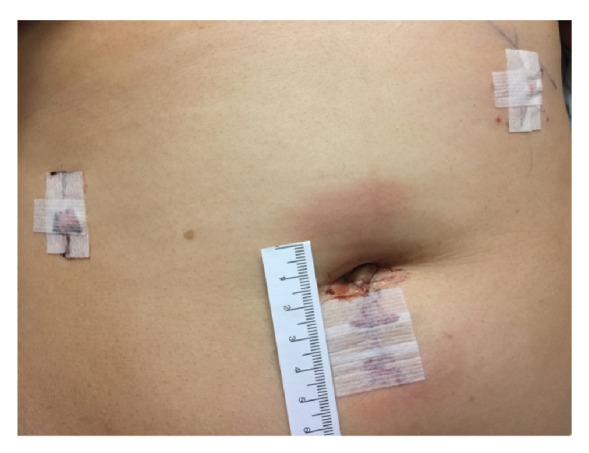
One 3 cm mini-laparotomic incision was made to hand-morcellate the large tumor.

Abdominal access was achieved with a left upper quadrant 5mm optical trocar and insufflation, followed by placement of a 10mm umbilical trocar and a right lateral 5 mm cannula. Abdominal exploration immediately showed approximately 500 mL of hemoperitoneum, with both dark and bright red blood seen to the level of the upper abdomen. There did not appear to be any ascites otherwise. No injury to the large vessels beneath the trocars was noted, but there was some confusion as to where the bleeding had arisen from. The mass was clearly seen, and with further manipulation, the uterus and left adnexa were noted to be completely normal. The right adnexa contained a smooth, hard right ovarian mass about 20cm in diameter. No normal ovarian tissue was identifiable. On further inspection, brisk bleeding was noted from the right ovarian artery of the right (IPL). The IPL was quickly clamped, which achieved hemostasis. The decision was made to perform a salpingo-oophorectomy, and this was performed with a Ligasure device. The umbilical incision (Figure 1) was extended to 3 cm, a medium sized Alexis retractor was placed, and the mass was hand morcellated. The mass was very hard, and many calcified areas were noted while morcellating. A biopsy was sent immediately after extending the incision and before continuing, and the frozen pathological diagnosis was ovarian fibroma. Operating time from incision to closure was 50 minutes. The patient did not require a blood transfusion. Post-operative recovery was uncomplicated and a post-operative CBC showed that the hemoglobin was 11.9g/dL and the hematocrit was 34.6%. The patient was discharged after post-operative milestones were met. Final histopathologic examination revealed an ovarian fibroma weighing 1,093.00 grams.

## 4. Discussion

Symptoms and sequelae of fibromas include abdominal discomfort or pain because of ovarian and/or appendiceal torsion, and/or ascites [1, 4–6]. However, there are no publications associating abdominal bleeding with ovarian fibromas, making our case unique. It is unclear when the tear may have occurred, but likely the significant weight of the mass and its mobility predisposed to the tearing of the IPL. Given the volume of blood, the briskness of the bleeding, and the dark and bright red blood present, we surmise that the bleeding may have been occurring for hours. Interestingly, our patient had no significant abdominal pain on the day of surgery outside of her chronic baseline abdominal discomfort.

An important consideration in the differential diagnosis of this patient should be idiopathic spontaneous intraperitoneal haemorrhage (ISIH), or abdominal apoplexy, which is a rare and often fatal diagnosis. Increasing but still very limited reports mention that ISIH may result from a variety of disease processes affecting the arterial and venous abdominal vasculature, e.g., cardiovascular diseases and diabetes [7, 8]. Law et al. [7] reported that hypertension and abdominal aortic atherosclerosis may be the potential risk factors. Harbour et al. [8] reported two fatal cases autopsy diagnosed with ISH, one involving arterial dissection of the gastroduodenal artery and the other one involving rupture of the superior mesenteric-portal venous system. Samina et al. [9] reported one case without any pre-, intra-, or post- operation risk factors being identified. Under such situations, preoperative and intraoperative diagnosis and treatment of abdominal apoplexy are extremely challenging. In our case, the patient had no risk factors and the bleeding clearly originated from the IPL. We believe the heavy ovarian fibroma played a role in tearing the IPL. However, a true idiopathic spontaneous hemorrhage should be always evaluated in a multidisciplinary context in conjunction with a gynecologic evaluation.

The diagnosis of an ovarian fibroma is difficult and the tumor is not often diagnosed accurately until the time of surgery and/or pathological diagnosis. There is a lack of characteristic symptoms and ultrasound and even MRI cannot easily distinguish an ovarian fibroma from uterine leiomyoma or other types of ovarian masses. It has been reported that 34% of ovarian fibromas were misdiagnosed preoperatively as a uterine fibroid [1, 4]. Up to 67% of these patients suffered from ascites, even with small fibromas [1, 4]. High levels of serum CA-125 in many of these cases may frequently cause a preoperative misdiagnosis of malignant ovarian neoplasia [1, 4]. Given this information, it is imperative to include ovarian fibroma as part of a differential diagnosis when faced with a solid pelvic mass, and preoperative counselling should include this possibility as well.

**Figure 2:**
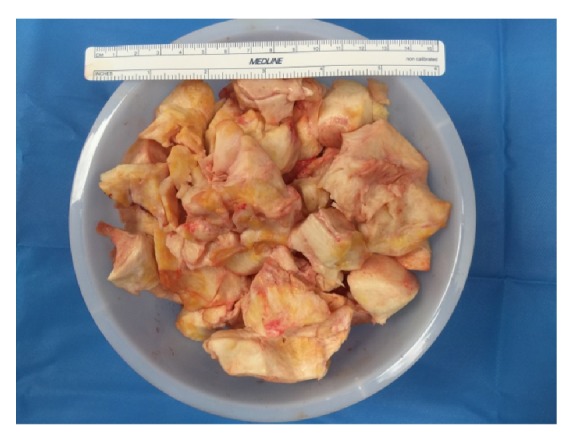
Ovarian fibroma, 48 ounce container. The mass was very hard, and many calcified areas were noted while morcellating.

Surgery is the treatment for ovarian fibromas. Generally, salpingo-oophorectomy can be considered [1, 4, 5] in peri- or post-menopausal women, and cystectomy may be performed in youths if there is an identifiable ovarian tissue [1, 4]. Son EC et al. [4] reported only 5 cases of laparoscopic ovarian cystectomy in a retrospective review of 47 women with confirmed ovarian fibromas. In our 39-year-old patient, no identifiable normal ovarian tissue was noted, and given the IPL bleeding, we performed a right salpingo-oophorectomy. This procedure was done via laparoscopy and mini-laparotomy. Compared to laparotomic surgery, the laparoscopic approach has several advantages including shorter hospital stay, faster recover of bowel activities, faster return of social life, less morbidity and better cosmetic results [1, 4, 5, 10, 11]. The biggest concern for laparoscopic surgery may be the difficulty in removing the tumor. We chose to extend the umbilical incision vertically in case the frozen pathology returned as malignant. Thus, further extension of the incision would not be difficult. Our intra-operative suspicion for malignancy was very low and we obtained the frozen section benign diagnosis before continuing with morcellation. Morcellation in a long strip is often done, but is difficult or impossible with a similarly calcified mass. Therefore we removed the specimen in sections (Figure 2).

## 5. Conclusion

When counselling patients, it is important to mention that significant abdominal bleeding can be associated with ovarian fibromas. It is important to include fibromas in the differential of a solid pelvic mass, even if imaging has diagnosed a uterine leiomyoma. A minimally invasive approach is feasible despite the size and hardness encountered via laparoscopy and mini-laparotomy.
